# Identification of Human Dihydroorotate Dehydrogenase Inhibitor by a Pharmacophore-Based Virtual Screening Study

**DOI:** 10.3390/molecules27123660

**Published:** 2022-06-07

**Authors:** Salvatore Galati, Stefano Sainas, Marta Giorgis, Donatella Boschi, Marco L. Lolli, Gabriella Ortore, Giulio Poli, Tiziano Tuccinardi

**Affiliations:** 1 Department of Pharmacy, University of Pisa, 56126 Pisa, Italy; salvatore.galati@phd.unipi.it (S.G.); gabriella.ortore@unipi.it (G.O.); tiziano.tuccinardi@unipi.it (T.T.); 2 Department of Drug Science and Technology, University of Turin, 10125 Turin, Italy; stefano.sainas@unito.it (S.S.); marta.giorgis@unito.it (M.G.); donatella.boschi@unito.it (D.B.); marco.lolli@unito.it (M.L.L.)

**Keywords:** virtual screening, pharmacophore model, human dihydroorotate dehydrogenase

## Abstract

Human dihydroorotate dehydrogenase (hDHODH) is an enzyme belonging to a flavin mononucleotide (FMN)-dependent family involved in de novo pyrimidine biosynthesis, a key biological pathway for highly proliferating cancer cells and pathogens. In fact, hDHODH proved to be a promising therapeutic target for the treatment of acute myelogenous leukemia, multiple myeloma, and viral and bacterial infections; therefore, the identification of novel hDHODH ligands represents a hot topic in medicinal chemistry. In this work, we reported a virtual screening study for the identification of new promising hDHODH inhibitors. A pharmacophore-based approach combined with a consensus docking analysis and molecular dynamics simulations was applied to screen a large database of commercial compounds. The whole virtual screening protocol allowed for the identification of a novel compound that is endowed with promising inhibitory activity against hDHODH and is structurally different from known ligands. These results validated the reliability of the in silico workflow and provided a valuable starting point for hit-to-lead and future lead optimization studies aimed at the development of new potent hDHODH inhibitors.

## 1. Introduction

**Figure 1:**
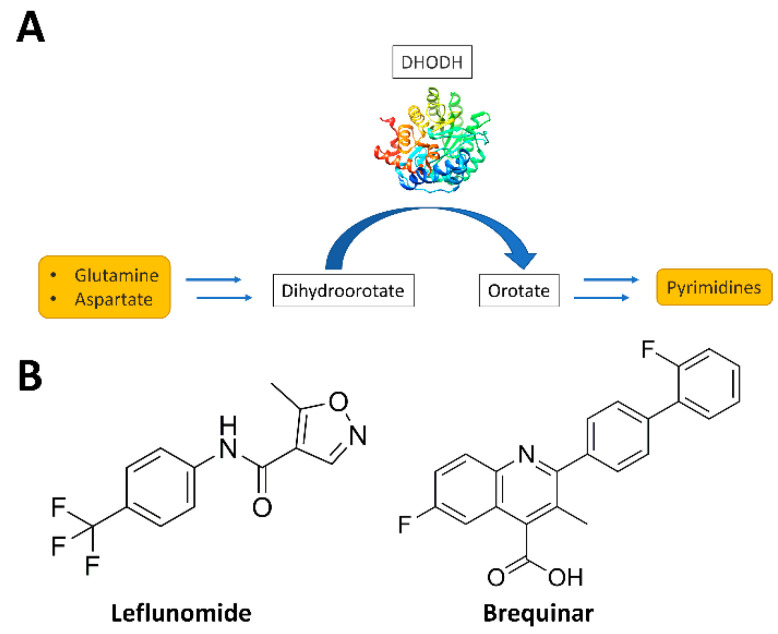
Scheme of the role of dihydroorotate dehydrogenase (DHODH) in de novo pyrimidine biosynthesis (**A**) and structures of the DHODH inhibitors leflunomide and brequinar (**B**).

Pyrimidine bases are required for the biosynthesis of DNA, RNA, glycoproteins, and phospholipids and therefore represent the basis of all biological activities. For this reason, rapidly proliferating cells, such as cancer cells, which have an increased demand for nucleic acid precursors and other cellular components, depend heavily on pyrimidine production. Furthermore, pyrimidines are essential for cell survival and the proliferation of living parasitic organisms, such as *Helicobacter pylori*, *Plasmodium falciparum*, and *Schistosoma mansoni*, that are able to impact upon human health. By consequence, the pyrimidine synthesis pathway has attracted particular interest in therapy. There are two possible ways by which pyrimidine synthesis can occur: the de novo synthesis pathway and the *salvage* pathway. The latter represents an effective method of recycling pre-existing nucleotides [1]. Many parasitic organisms lack salvage pathways for pyrimidine nucleotides and therefore are totally dependent on the de novo biosynthesis of pyrimidines, which thus represents an important element for selectively targeting the parasite without affecting the *human* host. The pyrimidine biosynthetic pathway is also remarkably active in highly proliferative cells such as cancer cells, thus representing a target for novel potential anticancer treatments. The key enzyme in the de novo pyrimidine nucleotides biosynthetic pathway is dihydroorotate dehydrogenase (DHODH) [2]. This flavin-dependent enzyme, located in the inner membrane of the mitochondria, is the only redox enzyme of the six enzymes involved in the de novo synthesis of pyrimidines. Indeed, DHODH catalyzes the conversion of dihydroorotate to orotate, an important precursor in the biosynthesis of pyrimidine bases (Figure 1), by transferring electrons via the involvement of two cofactors, flavin mononucleotide (FMN) and either nicotinamide adenine dinucleotide (NAD) or ubiquinone (CoQ) [3]. The inhibition of DHODH determines pyrimidine depletion and halt cell cycle progression at the S-phase, where a sufficient concentration of nucleotides is required for continued growth [4]. Pyrimidine depletion has been exploited to develop therapies for many diseases including viral and bacterial infections [5], parasitic diseases (i.e., malaria) [6], autoimmune disorders, and cancer [7]. In addition, recent works demonstrated the important role of *human* DHODH (*h*DHODH) in acute myelogenous leukemia (AML), a disease that affects the myeloid lineage of white blood cells by leading to accumulation in the bone marrow of immature cells and by interfering with the production of normal blood cells [8,9]. A variety of *h*DHODH inhibitors have been studied over the years—for example, leflunomide and brequinar (Figure 1).

Leflunomide, together with its metabolite teriflunomide, has been approved for the treatment of rheumatoid arthritis and other autoimmune diseases, and it is currently being evaluated as a single agent in clinical trials for multiple myeloma [10]. Brequinar is one of the most potent and well-known DHODH inhibitors, but it was unfortunately discarded as a therapeutic agent because it did not demonstrate widespread success in cancer clinical trials. Indeed, despite promising preclinical results, in the late 1980s and early 1990s, brequinar failed to generate a good response in multiple phase II clinical trials for solid tumors such as breast [11], colon [12], lung [13], and skin cancers [14]. Nevertheless, it is actually on clinical trial in acute myeloid leukaemia (NCT03760666) and in COVID-19 infections (NCT04575038). Therefore, the identification of novel *h*DHODH inhibitors is still a hot topic in the drug discovery field. In this work, a receptor-based virtual screening study employing pharmacophore modeling, consensus docking, and molecular dynamics (MD) simulations was carried out with the aim of discovering structurally novel *h*DHODH inhibitors. The whole workflow resulted in the identification of a novel compound endowed with micromolar inhibitory activity against *h*DHODH that can be considered as a valuable starting point for hit-to-lead and future lead optimization studies.

## 2. Materials and Methods

### 2.1. Pharmacophore Model Creation

LigandScout 4.4 [15] was employed to create the pharmacophore model. The pharmacophore hypothesis was built from the X-ray structure of *h*DHODH in complex with the 2-Hydroxypyrazolo[1,5-a]pyridine inhibitor named MEDS433 (PDB code: 6FMD) [16]. A complete model including all the possible pharmacophore features recognized by the software was generated, and, subsequently, only the desired features were retained in the final pharmacophore model. The four selected features included three H-bond acceptors and one aromatic feature, which were all set as mandatory during the pharmacophore screening. Furthermore, the excluded volume spheres generated on the basis of the receptor structure were added to the model. These latter features identify regions of space that cannot be occupied by ligands and thus provide a sort of steric filter during pharmacophore screening.

### 2.2. Database Generation and Pharmacophore Screening

Approximately 4 million compounds belonging to the Vitas-M, ChemBridge, Enamine, and Pharmeks commercial databases composed the initial screening library. The screening database was pre-filtered, retaining only compounds with at least one negatively charged group at the physiological pH. The pre-filter step was performed with the RDKit [17] library and resulted in 409,484 compounds. For the selected molecules, the conformational sampling and 3D database set-up was carried out with the iCon [18] software implemented in LigandScout, thus creating a LigandScout database for pharmacophore screening. The previously generated pharmacophore model including four mandatory features was used to screen the database in order to identify suitable compounds matching all the pharmacophore features.

### 2.3. Active Ligands Dataset Generation

The active ligands for *h*DHODH were extracted from the ChEMBL 30 database. The compounds whose inhibition potency was measured as K_i_, K_d_, and IC_50_ were selected. To obtain a unique data set of compounds, if multiple potency values were reported for a single molecule, the highest value was considered, and only compounds with K_i_/K_d_/IC_50_ below 100 µM were considered. The selected compounds were processed using an in-house python script with the aim of standardizing the chemical structures and removing salts. Subsequently, RDKit functions were used to apply the pre-filter to obtain only those compounds that presented at least one negatively charged moiety at the physiological pH. The ligand MEDS433, used to generate the pharmacophore, was removed from the final dataset, which comprised 524 molecules. Conformational sampling for the final dataset of active ligands was performed with the iCon software within LigandScout.

### 2.4. Molecular Docking Studies

The docking calculations were performed using the X-ray structure of *h*DHODH in complex with MEDS433 (PDB code: 6FMD). Thirteen different docking procedures were used in this study: AutoDock 4.2.3, Autodock Vina 1.1, Dock 6.7, Fred 3.0, GlamDock, Gold 5.1 (with its four fitness functions, i.e., ChemScore, GoldScore, ChemPLP, and Astex Statistical Potential), Glide 5.0 (with its standard precision (SP) and extra precision (XP) methods), Plants 1.2, and rDOCK, employing the procedures previously described [19,20]. Self-docking evaluations were performed for each docking method, using the rms_analysis tool within the Gold suite, by calculating the root-mean-square deviation (RMSD) between the position of the crystallized ligand predicted by the docking and its known experimental disposition.

### 2.5. Consensus Docking Evaluation

For each compound, 13 different binding poses were obtained (considering the top-scored pose for each docking method). The RMSD of each docking pose relative to the remaining docking dispositions was calculated through the rms_analysis software from the Gold suite, and a 13 × 13 matrix was generated, reporting the RMSD results. With the application of an in-house python script, the docking poses of each ligand were clustered to identify similar docking poses. The full linkage method was used as a hierarchical clustering algorithm for the grouping of the poses, using an RMSD cut-off of 2.0 Å, thus generating clusters of poses with mutual distances in terms of RMSD values of less than 2.0 Å. For each ligand, the consensus level was defined as the number of docking poses that were clustered within the RMSD cut-off of 2.0 Å and, consequently, as the number of docking methods that generated similar binding poses.

### 2.6. Molecular Dynamics Simulations

All of the simulations were performed using AMBER, version 20. General amber force field (GAFF) parameters were assigned to the ligands, while partial charges were determined using the AM1-BCC method, as implemented in the Antechamber suite. Ligand–protein complexes were placed in a rectangular parallelepiped water-box by using the TIP3P explicit solvent model and were solvated by employing a minimum distance of 15 angstroms of water between the protein and the sides of the box. Chlorine and sodium ions were added as counterions in order to neutralize the systems. Before the MD simulations, two stages of minimization were carried out; in the first step, a position restraint of 100 kcal/(mol·Å^2^) was applied to the complexes, thus minimizing only the position of the water molecules through 5000 steps of the steepest descent followed by the conjugate gradient until a convergence of 0.05 kcal/(mol·Å^2^). Successively, the whole system was energy minimized, imposing a harmonic force constant of 10 kcal/(mol·Å^2^) only on the protein α carbons. Periodic boundary conditions and Particle Mesh Ewald (PME) electrostatics were employed in the simulations. The minimized complexes were used as the starting conformations for the MD simulations. The time step of the simulations was 2.0 fs, a cut-off of 10.0 Å was set for the non-bonded interactions, and the SHAKE algorithm was used to keep all the bonds involving hydrogen atoms rigid. A constant-volume MD simulation was performed for the first 0.5 ns, during which the temperature of the systems was raised from 0 to 300 K. The systems were then equilibrated through 3 ns of constant pressure simulation, using the Langevin thermostat in order to maintain the temperature of the systems constant. Then, 46.5 ns of constant pressure periodic boundary MD was carried out at 300 K by using the Langevin thermostat. Hence, a total of 50 ns of MD simulation was carried out for each protein–ligand complex analyzed in this study. All the α carbons of the protein were restrained with a harmonic force constant of 10 kcal/mol·Å^2^ during the whole MD simulation. All the obtained MD trajectories were analyzed using the cpptraj program implemented in Amber 20.

### 2.7. hDHODH Inhibition Assay

The *h*DHODH inhibitory activity of the selected compounds was assessed by monitoring the reduction of 2,6-dichloroindophenol (DCIP), which is associated with the oxidation of dihydroorotate, as catalyzed by the DHODH enzyme. The human recombinant enzyme (Bio-Techne R&D Systems Inc.—Minneapolis, MN, USA) was preincubated for 5 min at 37 °C in a Tris-buffer (pH 8.0) solution with TritonX100 (final concentration 0.1% *v*/*v*), coenzyme Q10 (100 μM), and DCIP (50 μM), with the compounds that were to be tested used at 100 µM (final DMSO concentration 0.1% *v*/*v*). The reaction was initiated by the addition of dihydroorotate (500 μM), and the reduction was monitored at λ = 650 nm. The initial rate was measured in the first 5 min (ε = 10,400 M^−1^ cm^−1^), and the % inhibition was calculated using the GraphPad Prism 7 software. For one compound, different concentrations were analyzed and an IC_50_ value was calculated [21]. The values are the means ± SE of three independent experiments.

## 3. Results and Discussion

**Figure 2:**
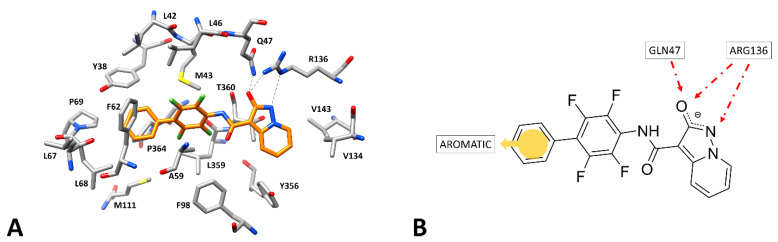
Hydroxypyrazolo[1,5-a]pyridine inhibitor MEDS433 within the binding site of *h*DHODH (PDB code: 6FMD). (**A**) The ligand (orange) is surrounded by the protein residues that constitute the binding site (shown as gray sticks), while the hydrogen bonds are shown as black dashed lines. (**B**) Receptor-based pharmacophore model with the four features superimposed to the *h*DHODH inhibitor structure.

With the aim of identifying novel inhibitors of *h*DHODH, we developed a receptor-based virtual screening (VS) study, focusing the first step of our in silico strategy on the X-ray structure of *h*DHODH in complex with our previously developed Hydroxypyrazolo[1,5-a]pyridine inhibitor MEDS433 (PDB code: 6FMD) [22,23], which is endowed with low nanomolar activity against *h*DHODH (IC_50_ = 1.2 nM) and thus represents one of the most potent inhibitors reported in the literature. The crystallized complex provided insights into the fundamental interactions that are supposedly responsible for enzyme inhibition. The ligand is bound to the ubiquinone binding site, which includes an outer lipophilic portion and an inner hydrophilic region (Figure 2A). The outer lipophilic cavity, constituting the entrance of the protein binding site, is occupied by the tetrafluorobiphenyl moiety of the ligand; on the other hand, the Hydroxypyrazolo[1,5-a]pyridine moiety of the compound is located in the inner and hydrophilic portion of the binding site. The presence of a negative charge delocalized on the oxygen and nitrogen of the ligand Hydroxypyrazolo[1,5-a]pyridine fragment, a carboxylate bioisoster with a pK_a_ between 5 and 5.5 [16], allows for the formation of strong H-bond interactions, with the key anchoring residues of the enzyme, which are fundamental for the stability of the binding mode of the inhibitor, located in the hydrophilic cavity. Precisely, an H-bond between the ligand oxygen atom and the side chains of Q47 and R136 can be observed; moreover, a further H-bond with R136 is established through the pyrazole nitrogen of the ligand. The biphenyl moiety of the inhibitor fits well into the lipophilic portion of the protein binding site delimited by Y38, L42, M43, L46, A59, F62, L68, L359, and P364, forming hydrophobic interactions with most of these residues. In particular, the terminal phenyl ring of the ligand is sandwiched between P364 and F62, forming a T-shaped stacking with this latter residue, and also interacts with Y38 and L68, thus suitably occluding the entrance of the protein binding site. According to the protein–ligand interactions observed in the reference X-ray complex, a receptor-based pharmacophore model was constructed using LigandScout [15]. The pharmacophore model included a total of four features: two H-bond acceptors representing the interactions with Q47 and R136 side chains established by the negatively charged oxygen of the ligand, one further H-bond acceptor representing the additional interaction of the pyrazole nitrogen with the side chain of R136, and one aromatic feature representing the interactions formed by the terminal phenyl group with the lipophilic cavity residues (Figure 2B). Furthermore, the model was refined by the addition of exclusion volume spheres representing regions of space occupied by the protein residues surrounding the co-crystallized inhibitor, which therefore cannot be occupied by ligands during a pharmacophore screening.

A virtual library gathering more than 4 million commercially available compounds was used for the vs. study. The library was first pre-filtered, retaining only compounds with at least a negatively charged moiety at the physiological pH, which is thought to represent a fundamental feature for *h*DHODH inhibitory activity. This filtering resulted in 409,484 compounds.

With the aim of evaluating the reliability of the pharmacophore model for VS, we performed a validation test using known active ligands of *h*DHODH. A curated dataset including 524 active ligands with at least a negatively charged moiety at the physiological pH, retrieved from ChEMBL database, was generated (see Materials and Methods for details). These compounds were used to enrich the pre-filtered screening library comprising 409,484 commercial compounds, thus obtaining an initial dataset in which the known active ligands corresponded to 0.13% of the whole screening library (Table S1, Supplementary Materials). The pharmacophore model was used to filter the screening library in order to identify compounds with a suitable molecular structure and establish key interactions with the enzyme binding site. A total of 1486 commercial compounds and 151 active ligands matching all four pharmacophore features of the model, while respecting the excluded volume constraints, were identified. As reported in Table S1, the pharmacophore screening filter retained 29% of the known active ligands and only 0.36% of the commercial compounds; therefore, the fraction of active compounds in the screened dataset corresponded to 9.2%, showing an increase of almost two logarithmic units in the fraction of the active ligands compared with that observed in the initial screening library. These findings validate the capacity of our pharmacophore model to retain the known active ligands among the selected compounds. The 1486 commercial compounds obtained from virtual screening were thus selected to be subjected to a consensus docking approach. Our research group performed various studies focused on the combined use of multiple docking methods at the same time, known as consensus docking, demonstrating the power of this approach in statistically increasing the reliability of docking results and in allowing for the discrimination between active ligands and inactive compounds against many different target proteins. In fact, we previously applied consensus docking in many vs. studies that allowed for the identification of novel hit compounds that were active against different types of protein targets [24,25,26,27,28]. Based on these considerations, we aimed at applying a consensus docking strategy for the identification of novel *h*DHODH ligands. Initially, 13 different docking procedures were tested through self-docking studies using the reference X-ray complex in order to assess their ability to reproduce the binding pose of the reference Hydroxypyrazolo[1,5-a]pyridine inhibitor MEDS433. Table 1 shows the root mean square deviation (RMSD) between the binding poses predicted by the docking and the experimental disposition of the ligand. On the basis of the self-docking analysis, we decided to employ all of the docking procedures within the consensus docking studies, since all of them were able to reproduce the binding mode of the reference ligand with good reliability (RMSD below 1.5 Å), as well as the key ligand–protein interactions represented by the pharmacophore model.

The 1486 molecules selected by the pharmacophore filter were thus docked into the reference X-ray structure of *h*DHODH using the 13 different docking methods, obtaining 13 different binding poses for each ligand (for each docking procedure, the top-scored pose was taken into account). A consensus docking analysis was performed using an in-house python script allowing for the calculation of the reciprocal RMSDs among all the binding poses predicted for each ligand, which were then clustered in the search for common binding modes (see Materials and Methods for details). The docked ligands were then ranked according to the obtained consensus level (i.e., the number of docking methods producing common binding modes). The results obtained from the consensus docking analysis, reported in Table 2, showed that no ligand achieved full consensus among all the docking procedures, since a maximum consensus level of 10 was obtained for the compounds. These results are in agreement both with our previous retrospective consensus docking evaluations [24] and our prospective vs. studies based on consensus docking, in which we observed that, statistically, only a very small percentage of the analyzed compounds is able to reach a high consensus among many different docking procedures [25,26], and often no compound obtains the maximum consensus level achievable [27,28]. Based on the results produced by the consensus docking protocol herein reported, 18 molecules showing at least a consensus level of 9 were considered for further studies.

The compounds selected through the consensus docking approach were then superimposed to the pharmacophore model in order to check whether their predicted binding mode still matched the four features of the model. Based on this post-docking filter, 5 out of 18 compounds that did not respect all of the pharmacophore features were discarded. The 13 remaining molecules were subjected to MD simulations aimed at assessing the stability of their predicted binding modes and key interactions with the residues of the enzyme binding site. Specifically, the 13 ligand–protein complexes were studied through 50 ns of MD simulation, the results of which were then analyzed taking into account the RMSD of the ligand position during the simulation with respect to the initial coordinates, as well as the stability of key H-bonds with Q47 and R136. Only six compounds, showing an average RMSD during the MD below 2.0 Å and maintaining at least two fundamental H-bonds with Q47 and R136 for more than 70% of the MD, were considered as new potential *h*DHODH inhibitors (Table S2, Supplementary Materials). A similarity search performed for the six compounds (Table 3) against previously published *h*DHODH ligands revealed that CPD1 was already reported as a potent inhibitor of *h*DHODH. In detail, CPD1 was found to be a structural analogue of the well-known *h*DHODH inhibitor brequinar (Figure 1), with an IC_50_ value of 51 nM [29]. As we were genuinely not aware of the presence of this compound within the commercial database used for our vs. study, this result alone already demonstrated the reliability of our in silico protocol in discovering *h*DHODH ligands, as it was able to identify a compound with high inhibitory activity against *h*DHODH. For the remaining five compounds selected by our vs. protocol, the ligand-similarity analysis showed that no *h*DHODH inhibitor with a similarity score greater than 80 (a score of 100 implies that two compounds are identical) was already reported (see Materials and Methods for details). Based on these results, the five compounds CPD2–CPD6 were purchased and subjected to in vitro studies in order to evaluate their biological activity. The enzyme inhibition assays revealed appreciable *h*DHODH inhibitory activity for two out of the five compounds at the concentration of 100 μM (Table 3). In particular, CPD5 showed the highest activity and was thus subjected to further evaluations, revealing a promising IC_50_ value for *h*DHODH inhibition (48 ± 8 μM).

**Figure 3:**
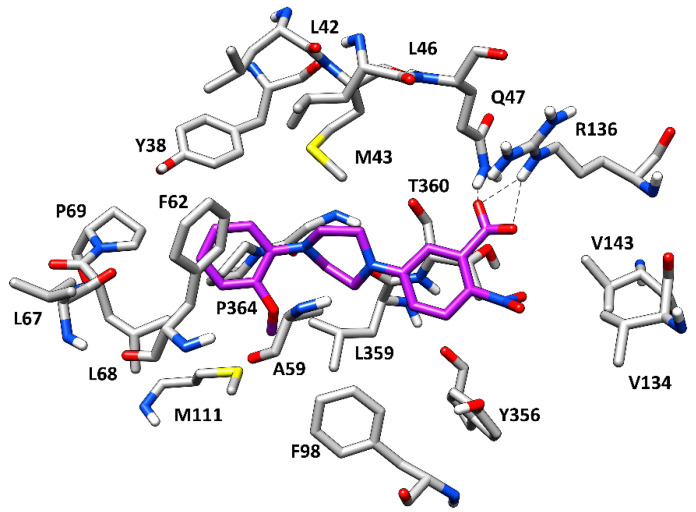
Minimized average structure of the compound CPD5 (purple) in complex with *h*DHODH. The protein residues surrounding the ligands, constituting the binding site, are shown as grey sticks, while the hydrogen bonds are shown as black dashed lines.

The predicted binding mode of CPD5 refined through MD simulations is shown in Figure 3. As expected, the negatively charged carboxylic group of the ligand was predicted to be placed within the inner hydrophilic cavity of the enzyme binding site. In particular, this moiety of the ligand perfectly mimics the negatively charged hydroxypyrazole portion of the reference inhibitor (Figure 2), forming strong H-bond interactions with the side chains of Q47 and R136 that were maintained for almost the entire MD simulation, suggesting their high importance for the binding stability of the compound. The aromatic ring connected to the carboxylic group of the compound primarily forms hydrophobic interactions with T360 and Y356, while the adjacent piperazine moiety is surrounded by M43, A59, and L359 and appears to establish van der Waals interactions with these residues. Lastly, the terminal anisolic moiety of the ligand occupies the outer lipophilic pocket of the binding site. In detail, the aromatic ring forms a T-shaped stacking with the phenyl ring of F62, as observed for the reference crystallographic inhibitor (Figure 2), and also shows lipophilic interactions with L68 and P364. Furthermore, the methoxyl group appears to establish additional hydrophobic interactions with the side chain of M111, thus further stabilizing the orientation of the aromatic ring into the hydrophobic pocket. Finally, the nitro group adjacent to the carboxylic moiety of the ligand does not appear to form relevant interactions with the protein binding site and may thus be profitably substituted with different groups in future hit-to-lead optimization studies aimed at improving the inhibitory potency of the compound.

## 4. Conclusions

Herein, we report a pharmacophore-based vs. study performed to identify novel inhibitors of *h*DHODH, which is gaining more and more attention within the medicinal chemistry community as a promising therapeutic target for the treatment of acute myelogenous leukemia, cancer, and viral and bacterial infections. The vs. protocol was based on a pharmacophore screening combined with consensus docking and refined by molecular dynamics simulations. Our in silico strategy, which was applied to screen a database of about 4 million commercial compounds, was able to identify a highly potent *h*DHODH inhibitor (IC_50_ = 0.051 μM) already reported in the literature, which was included without our knowledge in the database, as well as structurally novel *h*DHODH ligands. Among these, CPD5 showed promising inhibitory activity, with an IC_50_ value in the micromolar range. The obtained results confirmed the reliability of our vs. protocol and allowed for the identification of the novel *h*DHODH inhibitor CPD5, which is structurally different from the known ligands reported in the literature and which represents a valuable starting point for hit-to-lead and future lead optimization studies aimed at developing new potent *h*DHODH inhibitors.

## Figures and Tables

**Table 1:** RMSD results obtained with self-docking studies.

Docking Software	RMSD (Å)
rDock	1.2
Gold PLP	0.3
Gold Chemscore	0.7
Gold ASP	0.4
Gold Goldscore	0.6
Plants	0.4
Glide SP	0.2
Glide XP	0.2
Fred	0.9
Dock 6	0.2
Autodock	0.6
Glamdock	0.5
Vina	0.4

**Table 2:** Consensus docking results.

Consensus Level	No. of Compounds
13	0
12	0
11	0
10	7
9	11
8	22
7	37
6	47
5	76
4	79
3	320
2	721
1	166

**Table 3:** Structure and *h*DHODH inhibition activity of the selected compounds.

Structure	Compounds ID	% Inhibition (100 μM)	IC_50_ (μM)
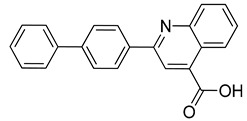	CPD1	n.d	0.051 ± 0.027 ^a^
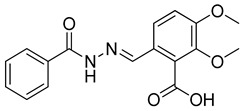	CPD2	8%	n.d
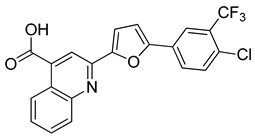	CPD3	48%	n.d
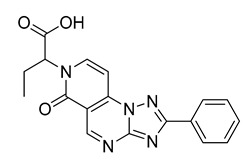	CPD4	16%	n.d
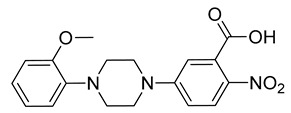	CPD5	72%	48 ± 8
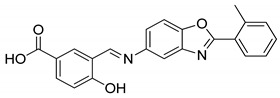	CPD6	0%	n.d

^a^ Potency value reported in the literature [29]. n.d: not determined.

## Data Availability

Data is contained within the article or Supplementary Materials.

## Supplementary Materials

The following supporting information can be downloaded at: https://www.mdpi.com/article/10.3390/molecules27123660/s1, Table S1: Validation statistics of the pharmacophore screening; Table S2: MD simulation results for the six analyzed ligand-*h*DHODH complexes.

## Sample Availability

Samples of the compounds are not available from the authors.
