# Supplementary material for: Identification of Human Dihydroorotate Dehydrogenase Inhibitor by a Pharmacophore-Based Virtual Screening Study
Source: Molecules. 2022 Jun 7;27(12):3660. doi: 10.3390/molecules27123660 (PMC9228440; doi:10.3390/molecules27123660)
Supplement: Supplementary file 1 [file molecules-27-03660-s001.zip › molecules-1742366-supplementary.pdf]

Supporting Information

Identification of Human Dihydroorotate Dehydrogenase Inhibitor by a Pharmacophore-Based Virtual Screening Study

Salvatore Galati [1], Stefano Sainas [2], Marta Giorgis [2], Donatella Boschi [2], Marco L. Lolli [2], Gabriella Ortore [1], Giulio Poli [1,*] and Tiziano Tuccinardi [1]

[1] Department of Pharmacy, University of Pisa, Pisa, Italy

[2] Department of Drug Science and Technology, University of Turin, Via P. Giuria 9, 10125 - Turin, Italy

*Correspondence: giulio.poli@unipi.it; Tel.: +39-0502219603

Table of Contents

Table S1. Validation statistics of the pharmacophore screening. S2

Table S2. MD simulation results for the six analyzed ligand-*h*DHODH complexes. S3

Table S1. Validation statistics of the pharmacophore screening, showing the fraction of actives in both the initial VS library and in the subset of compounds selected by pharmacophore screening.

	Commercial compounds	Active ligands	% actives
Initial pre-filtered compounds library	409484	524	0.13
Pharmacophore screening results	1486	151	9.2
Retained compounds (%)	0.36	29	

Table S2. Ligand RMSD and H-bond analysis during the MD simulation of the six analyzed ligand-*h*DHODH complexes.

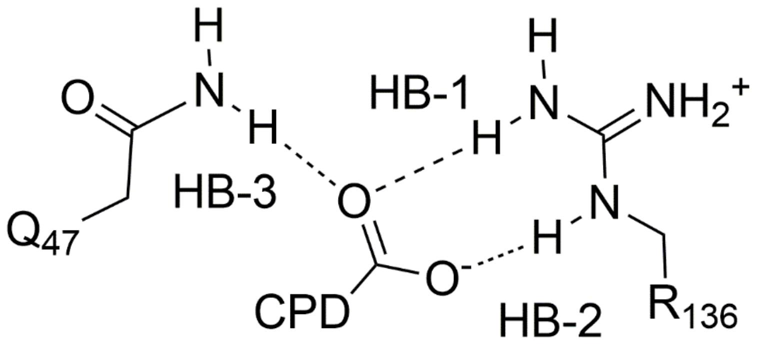

Compound	Average RMSD (Å)	HB-1 (%)	HB-2 (%)	HB-3 (%)
CPD1	0.7	100	100	83
CPD2	1.0	100	100	67
CPD3	0.8	100	100	45
CPD4	0.4	100	83	80
CPD5	0.7	100	100	81
CPD6	0.5	78	90	46
